# Clinical effectiveness of a rehabilitation program integrating exercise, self-management, and active coping strategies for chronic knee pain: A cluster randomized trial

**DOI:** 10.1002/art.22995

**Published:** 2007-10-15

**Authors:** M V Hurley, N E Walsh, H L Mitchell, T J Pimm, A Patel, E Williamson, R H Jones, P A Dieppe, B C Reeves

**Affiliations:** 1 King's College LondonLondon, UK; 2 University of the West of EnglandBristol, UK; 3 DeMontfort UniversityLeicester, UK; 4 Buckinghamshire Hospitals NHS TrustAylesbury, UK; 5 London School of Hygiene and Tropical MedicineLondon, UK; 6 MRC Health Services Research Collaboration, University of BristolBristol, UK; 7 University of BristolBristol, UK

**Keywords:** Integrated rehabilitation, Knee pain

## Abstract

**Objective:**

Chronic knee pain is a major cause of disability and health care expenditure, but there are concerns about efficacy, cost, and side effects associated with usual primary care. Conservative rehabilitation may offer a safe, effective, affordable alternative. We compared the effectiveness of a rehabilitation program integrating exercise, self-management, and active coping strategies (Enabling Self-management and Coping with Arthritic Knee Pain through Exercise [ESCAPE-knee pain]) with usual primary care in improving functioning in persons with chronic knee pain.

**Methods:**

We conducted a single-blind, pragmatic, cluster randomized controlled trial. Participants age ≥50 years, reporting knee pain for >6 months, were recruited from 54 inner-city primary care practices. Primary care practices were randomized to continued usual primary care (i.e., whatever intervention a participant's primary care physician deemed appropriate), usual primary care plus the rehabilitation program delivered to individual participants, or usual primary care plus the rehabilitation program delivered to groups of 8 participants. The primary outcome was self-reported functioning (Western Ontario and McMaster Universities Osteoarthritis Index physical functioning [WOMAC-func]) 6 months after completing rehabilitation.

**Results:**

A total of 418 participants were recruited; 76 (18%) withdrew, only 5 (1%) due to adverse events. Rehabilitated participants had better functioning than participants continuing usual primary care (−3.33 difference in WOMAC-func score; 95% confidence interval [95% CI] −5.88, −0.78; *P* = 0.01). Improvements were similar whether participants received individual rehabilitation (−3.53; 95% CI −6.52, −0.55) or group rehabilitation (−3.16; 95% CI −6.55, −0.12).

**Conclusion:**

ESCAPE-knee pain provides a safe, relatively brief intervention for chronic knee pain that is equally effective whether delivered to individuals or groups of participants.

## INTRODUCTION

Chronic knee pain is regarded as a mundane, inevitable, unmanageable consequence of aging. This overlooks the suffering, physical disability (1–4), psychosocial distress (5), health care expenditure (6–8), and socioeconomic burden (9) caused by chronic knee pain, and its indirect role through mobility and function impairment in the development of common chronic comorbidities (hypertension, diabetes, etc.) (10).

Primary care physicians label chronic knee pain as osteoarthritis (OA) and prescribe medication to relieve pain (1,11,12), but there are concerns about the safety (13,14), efficacy (15), and costs (16) of medication, particularly nonsteroidal antiinflammatory drugs (13,14). Furthermore, palliative medication does not address functional impairment (1–4). Improving function is best achieved if patients experience the benefits attainable from exercise (17–19) and patient education/self-management interventions (20–22). These interventions are usually delivered separately, but self-management interventions that do not include a significant exercise component are of limited value (22,23). Combining exercise and self-management might enhance their separate benefits, but few people will benefit if this produces complex, unworkable rehabilitation programs. As more people live longer (24) and patterns of incidence change (25), safe, effective, and efficient interventions that improve functioning and can be delivered to the large numbers of people will be needed.

We devised a brief rehabilitation program integrating a simple, personalized, progressive exercise regimen with patient education, self-management, and active coping strategies: Enabling Self-management and Coping with Arthritic Knee Pain through Exercise (ESCAPE-knee pain). Our primary hypothesis was that participation in ESCAPE-knee pain would improve functioning better than continuing usual primary care. A subsidiary hypothesis was that rehabilitation would be equally effective whether delivered to individuals or groups of people.

## PATIENTS AND METHODS

### Design

This pragmatic study compared usual primary care with a rehabilitation program designed to improve functioning using exercise, education, and self-management strategies to alter behavior and dispel inappropriate health beliefs according to a prespecified protocol. A pragmatic trial is carried out in a clinical setting and evaluates a feasible intervention for that setting using broad inclusion criteria to recruit a representative sample of the reference population, with few exclusion criteria to avoid maximizing the size of treatment effect by excluding individuals unlikely to benefit.

A cluster randomized trial design was chosen to minimize the risk of patients allocated to different arms exchanging information or primary care physicians altering their usual care (26,27). Primary care practices were the unit of randomization. The randomization list was generated at a central location away from the research center by an author (BR), who was not involved in the execution of the trial. Collaborating practices gave written consent for investigators to identify potential participants from their databases. Practices were randomly allocated in blocks of 3 to receive 1) usual primary care (whatever intervention a participant's primary care physician considered to be required and appropriate), 2) usual primary care plus individual rehabilitation (Indiv-rehab), or 3) usual primary care plus rehabilitation in groups of ∼8 participants (Grp-rehab).

The study was carried out between August 2000 and October 2004 in South East London, UK, after approval by relevant local research ethics committees. All assessments were conducted in the Rehabilitation Research Unit, Dulwich Community Hospital and all rehabilitation sessions performed in the Physiotherapy Out-patient Department.

### Study population

Broad inclusion criteria were adopted. We recruited individuals age 50 years or older who had consulted a primary care physician for mild, moderate, or severe knee pain of >6 months' duration. Many participants' condition had been labeled OA based on their clinical presentation and history without attempting to identify the cause of pain using investigations not routinely available to primary care physicians (e.g., radiographs). Exclusion criteria were as follows: lower limb arthroplasty, physiotherapy for knee pain in the preceding 12 months, intraarticular injections in the preceding 6 months, unstable medical conditions, inability/unwillingness to exercise, wheelchair dependence, and inability to understand English. Participants were not excluded if they used assistive walking devices; had stable comorbidities common in this age group (e.g., type II diabetes, cardiovascular or respiratory disorders); or had back, lower, or upper limb pain.

The (cluster randomized) design meant potential participants were given specific written information detailing the intervention they would receive. Persons interested in participating telephoned the investigators and received a verbal explanation of the trial; eligibility criteria were checked and a baseline assessment was arranged for those who were eligible and willing. At this assessment, written consent was obtained and a medical history taken. Management of all participants' knee and coexistent medical problems continued at the primary care physician's discretion and was documented at all assessments.

### Intervention

The rehabilitation program is outlined in Appendix A (available at the *Arthritis Care & Research* Web site at http://www.interscience.wiley.com/jpages/0004-3591:1/suppmat/index.html) with a detailed description available at www.kcl.ac.uk/gppc/escape. The content and format were identical for both Indiv-rehab and Grp-rehab, involving 12 supervised sessions (twice weekly for 6 weeks) that combined discussion on specific topics regarding self-management and coping, etc., with an individualized, progressive exercise regimen. To ensure consistency in content and delivery, the same experienced physiotherapist devised, supervised, and progressed all sessions for all participants (NW).

### Outcomes

The Likert version of the Western Ontario and McMaster Universities Osteoarthritis Index (WOMAC) was administered at each study visit. This questionnaire produces a total score (WOMAC-total, 0–96 points), with subscores for physical functioning (WOMAC-func, 0–68 points), pain (WOMAC-pain, 0–20 points), and stiffness (0–8 points, data not reported) (28). The WOMAC was completed by the participants. Lower WOMAC scores indicate better health status.

Secondary outcomes were pain (WOMAC-pain), objective functional performance (aggregated functional performance time of 4 common activities of daily living [AFPT]) (29), exercise health beliefs and self-efficacy questionnaire (ExBeliefs) (30), anxiety and depression (Hospital Anxiety and Depression Scale [HADS]) (31), self-reported health status (EuroQol) (32) converted into quality-adjusted life years based on utility weights collected in a UK general population sample (33), condition-specific patient preference health-related quality of life questionnaire (McMaster Toronto Arthritis [MACTAR]) (34), quadriceps strength (quadriceps maximum voluntary contraction), and quadriceps voluntary activation (29). Lower AFPT and HADS values and higher values in other outcomes indicate better health status. Assessment time was ∼45 minutes.

All outcomes were assessed at baseline, immediately after completion of the intervention or recruitment to the usual primary care arm (6-week assessment), and 6 months after completion of rehabilitation or 7.5 months after recruitment to the usual care arm, the prespecified primary end point. The immediate effect of the intervention (6-week assessment) on functioning is provided for comparison with other studies.

### Blinding

Outcome assessors were blinded to a participant's allocation. Success of blinding was evaluated by asking assessors to identify each participant's allocation at each assessment; if they identified rehabilitation they were further asked to identify whether the participant had been in the Indiv-rehab or Grp-rehab arm.

### Attendance

**Figure 1:**
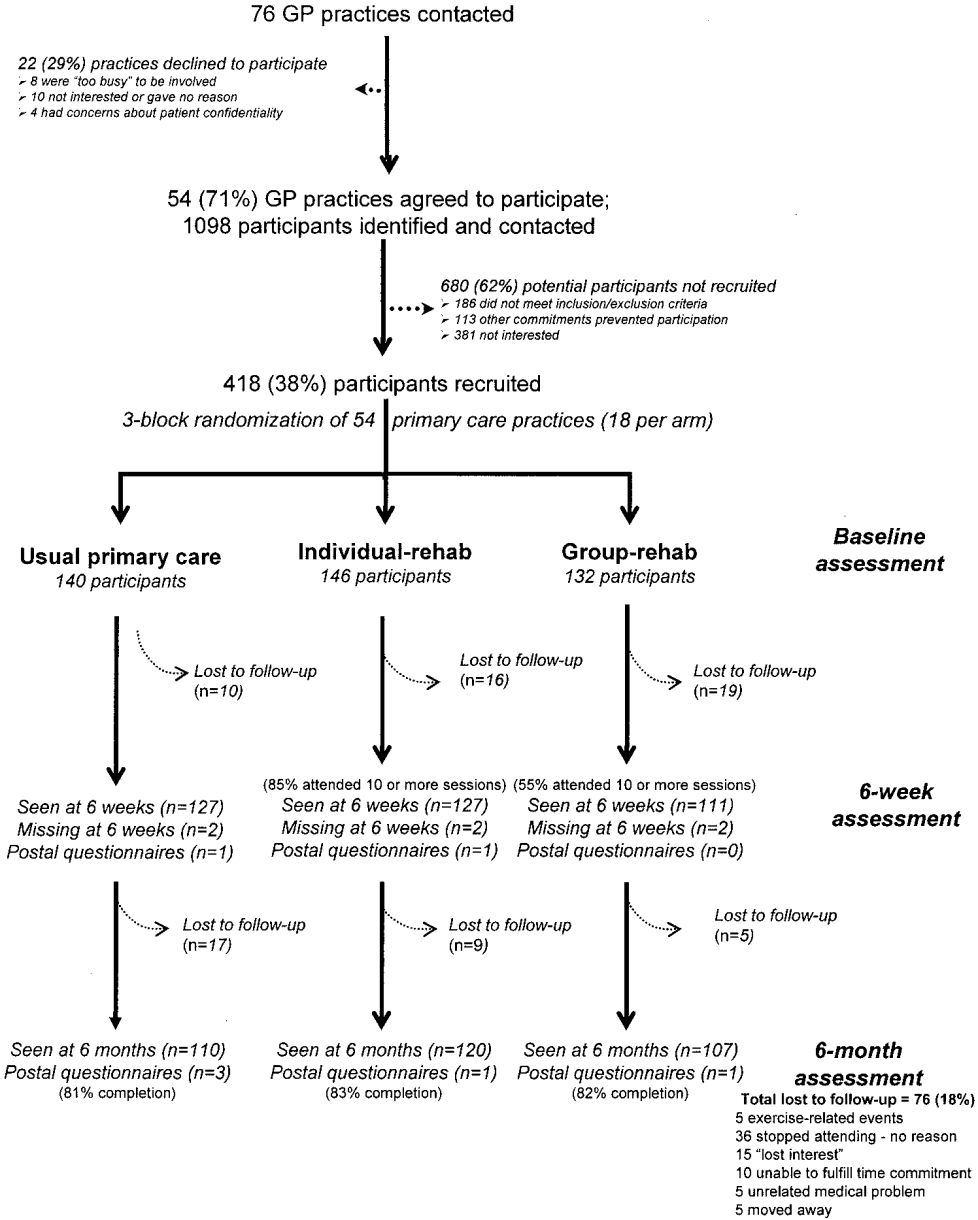
Flowchart of recruitment and retention of primary care practices and trial participants. GP = general practitioner.

Nonattendance at the supervised rehabilitation sessions was carefully recorded. Regardless of the number of rehabilitation sessions attended, all participants were invited to attend all assessments, or complete the outcome questionnaires by mail (n = 7) (Figure 1).

### Sample size

Lack of cluster randomized trials of chronic knee pain in primary care made sample size estimation problematic. A clinically meaningful difference was considered to be 15%. Based on the study by Bellamy et al of persons with knee OA who had a mean ± SD baseline WOMAC-func score of 41.3 ± 14.8 (28), we estimated that individual randomization would require 150 participants per arm for a trial with 90% power to detect a 15% difference between trial arms, with a 5% significance level (2-tailed) and allowing for 20% withdrawal by 6 months. Using intracluster correlation coefficients (ICCs) observed in other studies of chronic conditions in primary care (27), we inflated this sample size by 33% (i.e., a design effect of 1.33; 200 participants per arm) to take into account cluster randomization, and aimed to minimize the design effect by recruiting as many clusters as possible to decrease the average number of participants per cluster (26,27).

### Statistical analysis

Statistical analysis followed a prespecified protocol, based on intent-to-treat with no interim or post hoc analyses. Statistical significance was set at *P* less than 0.05. Outcomes for the 3 trial arms are presented as the mean ± SD or median (range), and standardized effect sizes (95% confidence intervals [95% CIs]) were calculated from the difference between the mean outcome at 6 months for one group minus the mean outcome at 6 months for another divided by the SD of the mean outcomes adjusted for baseline outcomes.

Because primary care practice characteristics did not affect the results, and the interventions were applied to individuals rather than primary care practices, demographic and clinical outcome variables are described for individual participants. To adjust for correlations between patients within a cluster, weighted *t*-tests or adjusted chi-square tests were used to test for differences between arms at baseline.

Multilevel modeling was used to estimate the group means and differences in outcome effect of the rehabilitation programs (using restricted iterative generalized least squares estimation using Multilevel Modelling for Windows, version 2.01, Institute of Education, London). Multilevel modeling was used to adjust for the intracluster correlations (patients within a primary care practice are likely to be more similar to one another than patients from another primary care practice), which violates the assumption of independence that is fundamental for the correct application of many statistical procedures.

Likelihood ratio tests were used to test random effects and Wald's test was used to test fixed parameters. WOMAC-func scores were initially compared across all 3 treatment arms, then for usual care versus rehabilitation (Indiv-rehab and Grp-rehab arms combined) and, pair-wise, each arm versus each other arm, using baseline WOMAC-func score as a covariate.

To estimate the effect of missing data, logistic regression was used to identify predictors of withdrawal. A longitudinal model was then constructed that jointly modeled the outcome at 6 weeks and the outcome at 6 months, allowing the outcome to be correlated for each participant. Variables associated with withdrawal were included in the model, allowing the effect of these variables to be different at 6 weeks and 6 months. This adjusts for the measured characteristics of participants who withdrew being different from those who remained in the trial. Missing data were assumed to be missing at random.

Preplanned interaction tests were performed to investigate whether treatment effects were influenced by anxiety and depression (HADS) or exercise health beliefs and confidence in ability to exercise (ExBeliefs). First, a model was fitted to 6-month WOMAC-func with the covariates baseline WOMAC-func, treatment group, and baseline HADS depression, followed by a further model with an interaction term of HADS depression with treatment group. This procedure was repeated using HADS anxiety, ExBeliefs total, and ExBeliefs self-efficacy.

For all comparisons, both unadjusted (adjusting only for the baseline covariate) and adjusted differences (adjusting for other potential confounding factors) were estimated, the latter as sensitivity analyses to explore the robustness of the unadjusted analyses to confounding. Because adjustment did not alter the effect size estimates, only the former are reported. Finally, the effect of unblinding, effect of assessor, and compliance with treatment were included in analyses to better interpret the effects of rehabilitation.

The number needed to treat (NNT; number of participants required to undertake rehabilitation so that 1 participant reports meaningful improvement in functioning [15%] 6 months after completion of rehabilitation) and 95% CI were calculated by comparing the proportion of rehabilitated participants who achieved this improvement compared with the proportion achieving the improvement with usual care.

## RESULTS

### Recruitment and retention

A total of 76 primary care practices were invited to collaborate; 22 declined or were unable to take part. Searches of the databases of the 54 consenting practices identified 1,098 potentially eligible participants who could be contacted. Of these, 186 (16%) were ineligible, 381 (34%) uninterested, and 113 (12%) unable to participate; 418 (38%) were recruited. The recruitment and retention of practices and individuals is shown in Figure 1. Although it was slightly more difficult to recruit participants into Grp-rehab, there was no evidence of differential recruitment to trial arms and all baseline characteristics were balanced (Table 1).

**Table 1:** Participant characteristics and mean baseline values for outcomes*

Variable	Usual care	Indiv-rehab	Grp-rehab
Sex, female:male	96:44	104:42	94:38
Age, mean (range) years	67 (51–89)	66 (50–91)	68 (51–84)
Duration of symptoms, median (IQR) years	6 (3–15)	7 (3–15)	5 (2.5–11)
Height, mean (range) meters	1.65 (1.46–1.89)	1.64 (1.45–1.97)	1.63 (1.39–1.92)
Body mass, mean (range) kg	81.8 (48–135)	80.6 (47–118)	80.2 (54–139)
Body mass index, mean (range) kg/m^2^	30.3 (20–51)	30.0 (18–45)	30.18 (20–50)
WOMAC-function	27.2 ± 14.6	26.4 ± 14.7	27.9 ± 14.7
WOMAC-pain	7.7 ± 4.0	7.4 ± 4.0	7.69 ± 4.1
WOMAC-total	38.6 ± 19.6	37.5 ± 19.4	39.1 ± 19.7
AFPT, seconds	66.0 ± 39.4	58.5 ± 29.9	65.7 ± 35.7
ExBeliefs self-efficacy	13.5 ± 3.5	14.1 ± 3.4	13.7 ± 3.2
ExBeliefs total	64.0 ± 7.7	65.0 ± 8.1	63.7 ± 7.7
HADS anxiety	6.7 ± 4.6	6.3 ± 3.9	6.6 ± 4.5
HADS depression	5.1 ± 3.7	4.5 ± 3.2	5.0 ± 3.4
MACTAR	31.6 ± 5.1	31.9 ± 5.0	31.2 ± 5.3
EQ-5D	0.60 ± 0.32	0.59 ± 0.28	0.60 ± 0.30
Right QMVC, newtons	238.4 ± 115	229.8 ± 102	214.7 ± 107
Right QVA, %	72.2 ± 23.6	71.1 ± 24.4	69.9 ± 25.6
Left QMVC, newtons	212.0 ± 106	202.2 ± 89	195.7 ± 95
Left QVA, %	71.9 ± 24.3	72.8 ± 24.4	72.9 ± 22.1

* Values are the mean ± SD unless otherwise indicated. Indiv-rehab = individual rehabilitation; Grp-rehab = group rehabilitation; IQR = interquartile range; WOMAC = Western Ontario and McMaster Universities Osteoarthritis Index; WOMAC-function = WOMAC function subscale; WOMAC-pain = WOMAC pain subscale; WOMAC-total = total score; AFPT = aggregate function performance time; ExBeliefs self-efficacy = exercise health beliefs self-efficacy subscale; ExBeliefs total = exercise health beliefs total score; HADS anxiety = Hospital Anxiety and Depression Scale anxiety subscale; HADS depression = Hospital Anxiety and Depression Scale depression subscale; MACTAR = McMasters Toronto Arthritis questionnaire; EQ-5D = EuroQol generic assessment of health status; QMVC = quadriceps maximum voluntary contraction; QVA = quadriceps voluntary activation.

By 6 months, 76 (18%) participants had withdrawn (Figure 1). There was no evidence of differential attrition. Only 5 (1%) participants withdrew because of exercise-related adverse events; 3 had exacerbation of pain (2 knee, 1 hip) and 2 with cardiac pacemakers had concerns about exercising, despite reassurance. No patient with outcome data was excluded from the analysis.

### Primary outcome

Mean baseline WOMAC-func was 27.2 (Table 2). Immediately after completing the program (6-week assessment), functioning improved (usual care 25.9, 95% CI 23.4, 28.3; rehabilitation 20.0, 95% CI 18.3, 21.7; Indiv-rehab 19.8, 95% CI 17.6, 22.0; Grp-rehab 20.2, 95% CI 17.6, 22.9). At the primary end point, 6 months after completing the program, some of these improvements had been lost but WOMAC-func differed significantly across the 3 arms (joint Wald's test, *P* = 0.04). WOMAC-func was better following rehabilitation compared with usual care (difference in WOMAC-func −3.33, 95% CI −5.88, −0.78, *P* = 0.01; effect size 0.29, 95% CI 0.07, 0.52; ICC 0.04) (Table 3).

**Table 2:** Estimated mean outcome (95% confidence intervals) at 6 months for usual primary care and rehabilitation groups, adjusting for baseline values*

		Outcome at 6 months	
Outcome	Baseline mean (n = 418)†	Usual primary care (n = 113)	Rehabilitation (n = 229)‡
Primary outcome			
WOMAC-function	27.2 (25.7, 28.6)	25.0 (22.9, 27.1)	21.6 (20.2, 23.1)
Secondary outcomes			
WOMAC-pain	7.6 (7.2, 8.0)	6.7 (6.1, 7.4)	5.7 (5.3, 6.2)
WOMAC-total	38.4 (36.5, 40.3)	35.0 (32.0, 38.0)	30.4 (28.3, 32.6)
AFPT, seconds	63.3 (59.8, 66.7)	61.0 (57.2, 64.9)	57.6 (54.9, 60.2)
ExBeliefs self-efficacy	13.8 (13.5, 14.1)	14.0 (13.4, 14.6)	15.3 (14.9, 15.7)
ExBeliefs total	64.2 (63.5, 65.0)	64.0 (62.7, 65.3)	67.5 (66.6, 68.5)
HADS anxiety	6.53 (6.11, 6.95)	5.97 (5.46, 6.49)	5.32 (4.96, 5.68)
HADS depression	4.86 (4.53, 5.20)	4.28 (3.87, 4.69)	3.93 (3.64, 4.22)
MACTAR	31.6 (31.1, 32.1)	41.8 (40.3, 43.3)	44.0 (42.9, 45.0)
EQ-5D	0.60 (0.57, 0.63)	0.66 (0.60, 0.71)	0.64 (0.61, 0.68)
Right QMVC, newtons	227.8 (216.5, 239.2)	230.2 (217.7, 242.7)	237.4 (228.8, 246.0)
Right QVA, %	71.0 (68.3, 73.6)	68.3 (61.8, 74.8)	74.7 (70.1, 79.4)
Left QMVC, newtons	203.4 (193.5, 213.6)	203.0 (187.6, 218.3)	210.8 (199.7, 221.9)
Left QVA, %	72.5 (70.0, 75.0)	70.6 (66.2, 75.0)	76.3 (73.3, 79.4)

* Estimates represent the predicted outcome for a participant with average baseline WOMAC-function, i.e., baseline WOMAC-function was centered around the mean before inclusion in the model. See Table 1 for definitions.

† Data from all participants.

‡ Number of participants analyzed for primary outcome for the individual and group rehabilitation arms are combined. The number analyzed for secondary outcomes was not always the same because a few data were missing for some outcomes.

**Table 3:** Effects of rehabilitation at 6 months (differences between rehabilitation and usual care, and 95% confidence intervals), adjusting for baseline values of outcomes*

Outcome	Effect size	Wald's test *P* value	Standardized effect size
Primary outcome			
WOMAC-function	−3.33 (−5.88, −0.78)	0.010	0.29 (0.07, 0.52)
Secondary outcomes			
WOMAC-pain	−1.01 (−1.84, −0.19)	0.016	0.27 (0.05, 0.50)
WOMAC-total	−4.59 (−8.30, −0.88)	0.015	0.28 (0.05, 0.50)
AFPT, seconds	−3.47 (−8.13, 1.19)	0.019	0.17 (−0.06, 0.41)
ExBeliefs self-efficacy	1.32 (0.58, 2.07)	0.0005	−0.41 (−0.63, −0.17)
ExBeliefs total	3.58 (2.00, 5.15)	< 0.0001	−0.51 (−0.75, −0.28)
HADS anxiety	−0.65 (−1.28, −0.02)	0.043	0.23 (0.01, 0.46)
HADS depression	−0.35 (−0.85, 0.16)	0.175	0.16 (−0.07, 0.38)
MACTAR	2.20 (0.36, 4.04)	0.019	−0.27 (−0.50, −0.04)
EQ-5D	−0.01 (−0.08, 0.05)	0.709	0.09 (−0.21, 0.39)
Right QMVC, newtons	7.18 (−8.04, 22.4)	0.355	−0.13 (−0.40, 0.14)
Right QVA, %	6.44 (−1.52, 14.4)	0.113	−0.20 (−0.44, 0.05)
Left QMVC, newtons	7.78 (−11.2, 26.8)	0.422	−0.10 (−0.34, 0.15)
Left QVA, %	5.71 (0.33, 11.1)	0.038	−0.26 (−0.51, −0.01)

* Differences are expressed in the units in which the outcome was measured. Standardized effect sizes estimated as Cohen's d. See Table 1 for definitions.

Mean WOMAC-func scores for the Indiv-rehab and Grp-rehab arms were significantly different from usual care (difference in WOMAC-func: Indiv-rehab −3.53, 95% CI −6.52, −0.55, *P* = 0.04; Grp-rehab −3.16, 95% CI −6.55, −0.12, *P* = 0.04) but not from each other (Indiv-rehab 21.5, 95% CI 19.3, 23.6; Grp-rehab 21.8, 95% CI 19.6, 24.0). NNT was 7 (95% CI 4, 27; calculation of NNTs from inversion of the relative risk causes asymmetric 95% CI). The odds ratio of benefiting from rehabilitation compared with usual care was 1.8 (95% CI 1.2, 2.8).

### Effect of withdrawal from trial

Participants who withdrew from the study between the 6-week and 6-month assessment had poorer functioning at 6 weeks than those who remained in the study (mean WOMAC-func 28.0 for dropouts versus 21.5 for non-dropouts; *P* = 0.013 for test of the difference). In particular, participants in the usual care arm who withdrew tended to have worse functioning at 6 weeks (mean WOMAC-func 34.8 for dropouts versus 24.5 for non-dropouts), whereas participants in Grp-rehab who withdrew tended to have better functioning (mean 6-week WOMAC-func 14.8 for dropouts versus 20.5 for non-dropouts). Adjusting for missing 6-week WOMAC-func scores improved the mean WOMAC-func to −6.45 (95% CI −12.5, −0.44) in Indiv-rehab and −6.14 (95% CI −11.6, −0.70) in Grp-rehab. These point estimates indicate a much greater effect than our primary analysis, with larger confidence intervals due to the added uncertainty in the model.

### Secondary outcomes

A similar pattern of results was found for the secondary outcomes (Table 2) and effect sizes (Table 3): there were differences between rehabilitation and usual care, but no difference between Indiv-rehab and Grp-rehab.

### Preplanned interaction tests

Baseline depression (HADS depression) had no influence on treatment effect but was a significant covariate, i.e., high baseline depression was associated with poor outcome (0.48, *P* = 0.011). Baseline positive exercise beliefs and confidence in the ability to exercise (ExBeliefs total) had no influence on treatment effect but was a significant covariate, i.e., participants with higher baseline ExBeliefs total scores had better functioning at 6 months (−0.24, *P* = 0.001). The same pattern of results was observed with greater confidence in ability to exercise (ExBeliefs self-efficacy), i.e., no interaction but better functioning at 6 months (−0.62, *P* = 0.001).

### Effect of assessor and blinding

Outcomes did not differ between the 2 assessors. Assessors correctly identified allocation in 133 (61%) of 219 participants in the rehabilitation groups. WOMAC-func was slightly, but not significantly, better in participants whom assessors identified as having received rehabilitation (difference in WOMAC-func −0.60; 95% CI −3.31, 2.11; *P* = 0.66). The effect size for the subgroup of rehabilitation participants whose allocation was not disclosed was smaller compared with usual care (difference in WOMAC-func −2.32; 95% CI −5.21, 0.57).

### Adherence

Of the participants who attended 6-month followup, 105 (85%) of 120 Indiv-rehab participants and 59 (55%) of 107 Grp-rehab participants attended ≥10 of the 12 sessions.

## DISCUSSION

For individuals with chronic knee pain, supplementing usual primary care with a personalized, progressive rehabilitation program integrating exercise, education, and active coping strategies (ESCAPE-knee pain) improved functioning for up to 6 months after completion of rehabilitation, regardless of whether it was delivered to individuals or small groups of patients. The major strengths of this trial are its rigor, size, realism, and applicability to less-controlled clinical contexts (35,36). This is the first pragmatic trial of efficacy for this type of complex health care intervention for chronic knee pain in primary care. It was designed, conducted, and analyzed according to a prespecified protocol, and had a high followup rate. The trial enrolled a representative inner-city population with common comorbidities that primary care physicians encounter in daily practice. Although there are no guidelines for primary care management of chronic knee pain, there are guidelines for management of OA, but these are poorly observed for many reasons (12,37). Our participants reported management typical of what has been reported in primary care (12,38,39): they used few health care resources and had disparate drug regimens with occasional referral (40,41). The rehabilitation program was carried out in a typical outpatient department. In addition, although its general content was similar, the specific exercise components, tailored to address individuals' needs, varied between participants and within participants over time as the exercises were progressed to remain challenging. These factors reflect usual clinical context and practice, improving the likelihood that the intervention and its effects will be replicated when performed in clinics (35,36).

Cluster randomized trials that admit individuals have an increased risk of selection bias and preferential recruitment (26). To avoid this, potential participants were identified from primary care practice registers before the practices were randomized. Potential participants were told the intervention they would receive before entering the trial, but because similar numbers of participants accepted, declined, or withdrew from all arms of the trial, preferential recruitment is unlikely to have affected the results. An advantage of the cluster design was that we could explain unambiguously to potential participants the intervention they would receive, avoiding the need to explain randomization procedures. We believe this facilitated recruitment and retention to a trial that required a significant commitment of time and effort by participants. Documenting the reasons for nonparticipation and withdrawal enables the generalizability of the findings to be assessed (35,36).

To standardize the program's content and delivery, one experienced physiotherapist delivered the program. This increases internal validity but might compromise generalizability if the effectiveness of the intervention were influenced by the therapist's personal qualities or experience. However, because ESCAPE-knee pain does not require specialized training, sophisticated exercises, equipment, or facilities, other therapists will find it easy to replicate.

Recruitment was slightly easier and attendance was better in Indiv-rehab than Grp-rehab. This was because individual rehabilitation sessions could be arranged at convenient times and missed sessions could be rearranged. Group sessions were scheduled at inflexible times that were sometimes inconvenient, and missed sessions could not be rearranged, which is consistent with clinical practice. Despite attendance differences, group and individual rehabilitation were equally effective, suggesting that the number of individual rehabilitation sessions might be reduced yet still provide an effective, convenient, flexible management option.

Blinding interventions that require active participation is difficult. Our assessors frequently identified allocation correctly based on what participants said or did not say, introducing assessor bias that could have affected outcome. A preplanned subgroup analysis suggests that unblinding was associated with slightly better outcome, but the difference was not statistically significant and the trial had little power to detect an important difference between subgroups, therefore this subgroup analysis must be interpreted cautiously. Because assessors could not distinguish between participants who had received individual or group rehabilitation, comparison of differences between these arms was not influenced by unblinding.

The trial did not achieve the target recruitment but nevertheless found significant differences between arms. This is largely because the sample size calculation did not take into account inclusion of baseline score as a covariate in the final analysis; doing so reduces sample size required for specified power almost by half (42), and recruiting many clusters with a relatively few average number of patients per practice minimized the design effect (26,27). Moreover, recent work suggests that 12% may represent meaningful change (43).

Participants with poor functioning in the usual care arm may have dropped out of the trial after becoming disillusioned with lack of additional intervention, while participants with good functioning in the intervention arm who experienced only small improvement may have also become disillusioned and dropped out. Conversely, participants with significant functioning deficits who experienced an effective intervention that involved time and effort on their part may have felt more committed to the trial. Regardless of the reason for dropping out of the trial, the differential withdrawal reduced the between-group differences, underestimating the intervention effect. Targeting individuals likely to benefit the most from the intervention might maximize efficient use of resources.

Our effect sizes for functioning (0.29; 95% CI 0.07, 0.52) and pain (0.27; 95% CI 0.05, 0.50) were similar to those found in meta-analyses of exercise (functioning effect size 0.32–0.45, pain effect size 0.39–0.52) (17–19), drug trials (functioning effect size 0.34, pain effect size 0.66) (21), and several recent studies of similar patient populations and interventions (23,44–46). However, most of these studies measured outcomes immediately after completing prolonged interventions. The improvements achieved during ESCAPE-knee pain were attained following a brief, clinically practicable intervention sustained for 6 months; the number needed to treat was much lower than in drug trials (47) but without adverse drug events.

Psychological variables and health beliefs are important determinants of functioning (48). In this study, participants with positive exercise beliefs who were confident in their ability to exercise had better functioning, whereas those with higher baseline depression had poorer outcome. People intuitively appreciate that movement is good for joints but associate movement with pain. In the absence of appropriate advice they become confused, frightened, and refrain from activities they believe may cause harm (49). ESCAPE-knee pain was designed to improve functioning, understanding, and confidence by combining education, advice, reassurance, and simple coping strategies with the experience of performing supervised functional activities and exercises. Successful performance of simple exercises and activities without painful exacerbation, coupled with information, advice, and reassurance from a knowledgeable health care professional, may have increased participants' understanding of the importance and benefit of physical activity, restored confidence in their abilities, and enabled them to appreciate how they could help themselves. In this respect the changes in exercise health beliefs and self-efficacy are notable. However, speculation about possible mechanisms needs to be interpreted cautiously until validated by studies specifically designed to elucidate the mechanisms of the intervention.

As the prevalence of chronic ill health increases, so does the need for safe, effective, low tech, affordable interventions that promote self-management and that can be delivered to large numbers of individuals (24). Such interventions permeate populations more effectively than complex, expensive interventions that may have large effects but that few people can access and benefit (35). Consequently, ESCAPE-knee pain could have an impact on management of knee pain: for the large and growing numbers of persons with chronic knee pain, ESCAPE-knee pain provides health benefits using active coping strategies that enable individuals to help themselves; for health care providers, ESCAPE-knee pain provides an effective, relatively brief, simple alternative or adjunct to medication without adverse side effects; for policymakers, ESCAPE-knee pain is an intervention that can be delivered to large numbers of individuals and that fits with health and social care policy of increasing physical activity and self-management for chronic conditions (50).

## AUTHOR CONTRIBUTIONS

Dr. Hurley had full access to all of the data in the study and takes responsibility for the integrity of the data and the accuracy of the data analysis.

**Study design.** Hurley, Walsh, Pimm, Patel, Jones, Dieppe, Reeves.

**Acquisition of data.** Hurley, Walsh, Mitchell.

**Analysis and interpretation of data.** Hurley, Walsh, Mitchell, Pimm, Williamson, Jones, Dieppe, Reeves.

**Manuscript preparation.** Hurley, Walsh, Mitchell, Pimm, Patel, Jones, Dieppe, Reeves.

**Statistical analysis.** Hurley, Williamson.

**Trial Steering Group members.** Hurley, Walsh, Mitchell, Pimm, Patel, Williamson, Jones, Dieppe, Reeves.
